# Human Papillomaviruses Target the DNA Damage Repair and Innate Immune Response Pathways to Allow for Persistent Infection

**DOI:** 10.3390/v13071390

**Published:** 2021-07-17

**Authors:** Elona Gusho, Laimonis Laimins

**Affiliations:** Department of Microbiology-Immunology, Feinberg School of Medicine, Northwestern University, Chicago, IL 60611, USA; elona.gusho@northwestern.edu

**Keywords:** DNA damage, innate immunity, HPVs, epithelial differentiation, DAMPs

## Abstract

Persistent infection with high-risk human papillomaviruses (HPVs) is the major risk factor associated with development of anogenital and oropharyngeal cancers. Initial infection by HPVs occurs into basal epithelial cells where viral genomes are established as nuclear episomes and persist until cleared by the immune response. Productive replication or amplification occurs upon differentiation and is dependent upon activation of the ataxia-telangiectasia mutated (ATM), ataxia telangiectasia and RAD3-related (ATR) DNA damage repair (DDR) pathways. In addition to activating DDR pathways, HPVs must escape innate immune surveillance mechanisms by antagonizing sensors, adaptors, interferons and antiviral gene expression. Both DDR and innate immune pathways are key host mechanisms that crosstalk with each other to maintain homeostasis of cells persistently infected with HPVs. Interestingly, it is still not fully understood why some HPV infections get cleared while others do not. Targeting of these two processes with antiviral therapies may provide opportunities for treatment of cancers caused by high-risk HPVs.

## 1. Introduction

Human papillomaviruses (HPVs) are important pathogens that are the causative agents of many anogenital as well as oral cancers [1]. Over 400 types of HPVs have been identified, and each infects stratified epithelia at different body locations. The viral types are denoted with a number according to the order of their discovery and generate a range of epithelial lesions. HPV type 1 infects epithelia in the soles of feet and causes plantar or palmar warts, while cutaneous HPVs (e.g., HPV 5) are responsible for warts on hands. About one-third of HPV types infect the genital epithelia and are classified as high and low risk according to their propensity to be associated with human cancers. At least 10 types are designated high risk and are the causative agents of over 98% of cervical cancers as well as those of the anus and vulva [2]. HPV16 is associated with approximately 50% of cases of cervical cancer, while HPV18 is detected in about 25% [1]. The remaining eight types are found in less than 5% each. In western countries, approximately 60% of oropharyngeal cancers are caused by HPV infection, and these are almost exclusively HPV16 positive [3]. Cases of oropharyngeal cancers are increasing at a rapid pace and likely will soon exceed those of cervical cancer. HPV-induced cancers typically take several decades to develop but can also regress spontaneously through activation of immune recognition. Low-risk HPVs also infect the genital epithelia (e.g., HPV 6, 11) but are rarely associated with cancer [4]. The incidence of HPV-induced cancers will decrease with time due to the development of prophylactic vaccines; however, these only block initial infection and have no effect on existing infections, which may still be able to progress to cancers. A need, therefore, exists to develop antivirals to treat cancers that develop in individuals, who are persistently infected. 

## 2. Genome Organization and Viral Proteins

HPV virions consist of an outer icosahedral shell that encapsidates its 7.9 kb circular double-stranded DNA genome that is bound by cellular histones. HPVs infect cells in the basal layer of stratified epithelia that become exposed by wounding. Following entry, viral genomes are established as nuclear episomes at about 100 copies per cell. These infected basal cells can persist until they are cleared by the immune system or progress to cancers that are often associated with the integration of viral DNAs into host chromosomes. In productive lesions, the synthesis of progeny virions, however, occurs in highly differentiated suprabasal cells [5,6]. 

**Figure 1:**
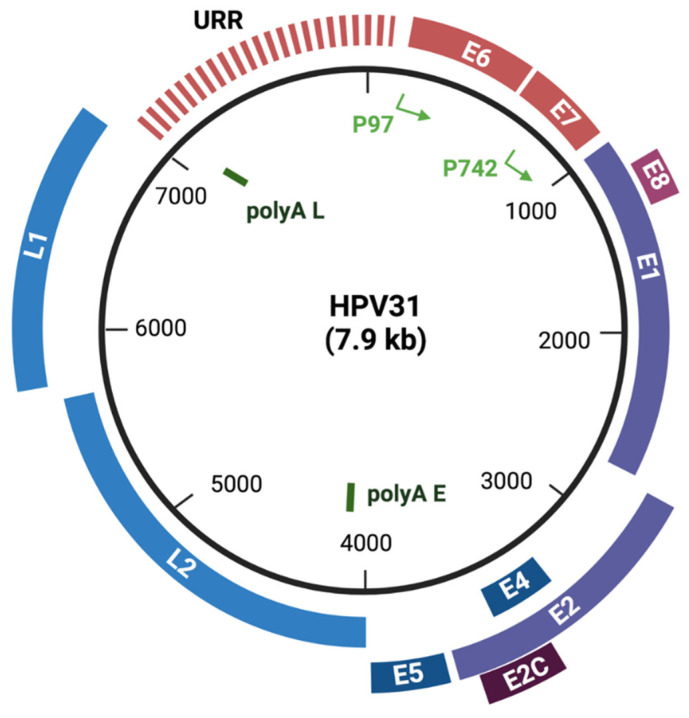
HPV genome structure map of high-risk HPV31. Viral genomes consist of two main coding regions: early (E) and late (L). The early (E) transcripts are initiated at the p97 promoter and are expressed in the lower portion of stratified epithelia. The E6 and E7 open reading frames encode oncogenes that target p53 and Rb, respectively, as well as other cellular proteins. E1 and E2 are the two replication proteins, while E4 and E5 function in the later stages of viral life cycle in suprabasal cells. E8^E2C is a repressor generated by splicing that regulates copy number. Late (L) gene transcription initiates upon differentiation from the p742 promoter and expresses high levels of E1^E4 as well as E5 along with the capsid proteins L1 and L2. The URR is a noncoding region harboring the origin of viral replication and enhancers to regulate viral promoters. HPV31 genome organization is similar to that of other high-risk HPVs such as HPV16 and 18. (Created with BioRender.com, 24 June 2021).

HPV genomes consist of two major coding regions along with a third regulatory region referred to alternatively as the upstream regulatory region (URR) or the long control region (LCR) (Figure 1). The early genes (E) are expressed prior to productive viral replication in suprabasal layers. About 5 to 8 early open reading frames are present in almost all HPV types and are expressed from polycistronic messages that primarily originate from a promoter upstream of the E6 ORF. Interestingly no monocistronic messages have been identified for HPVs indicating alternative splicing is a major regulator of viral expression. E6 and E7 act as viral oncoproteins, while E1 and E2 are replication proteins [7,8,9,10]. The E4 and E5 open reading frames are present in the early transcripts but are synthesized at high levels only following differentiation indicating that their primary function is during the late phase of the viral life cycle [11,12,13]. 

The E6 proteins are approximately 100 amino acids in size and are localized in both the cytoplasmic as well as nuclear compartments. The high-risk E6 proteins interact with a series of cellular factors that contribute to cellular transformation. The most prominent of these is ubiquitin ligase UBE3A (E6AP), which recruits p53 resulting in its rapid turnover [14,15]. High-risk E6 proteins also activate the catalytic subunit of telomerase, hTert, leading to its activation [16,17,18,19]. The extreme C-terminus of high-risk E6 proteins contain PDZ interaction domains, which allow for the binding of factors such as Scribble and DLG that are also important for transformation [20,21,22]. Low-risk E6 proteins do not induce degradation of p53 and instead interact with the MAML1 protein [23,24].

The E7 proteins are found primarily in the nucleus and bind to members of the Rb family of proteins. Binding of high-risk E7 protein induces degradation of Rb leading to constitutive activation of E2F family members, resulting in rapid entry into S-phase [25,26,27,28]. Low-risk E7 proteins also bind to RB proteins but with a significantly reduced affinity [29]. E7 proteins bind to p600 as well as HDACs among other factors [30,31,32].

The E1 proteins bind to HPV origin sequences and help to recruit cellular replication factors including DNA polymerases to viral genomes. E1 proteins also act as helicases to unwind strands of DNA ahead of replication complexes. Monomeric E1 proteins bind DNA with low affinity and form complexes with E2 proteins to facilitate their recruitment to HPV origins that are flanked by E2 binding sites [33,34,35,36,37]. Once recruited to origins they are assembled into stable hexameric structures, and E2 proteins are released [38,39]. In addition to their roles in replication, E2 proteins are both positive and negative regulators of viral transcription that is mediated through interactions with the bromodomain protein Brd4 [40,41,42]. The N-terminus of E2 contains a transactivation domain, while the C-terminus contains DNA binding sequences [43]. Truncated forms of E2 containing the C-terminus fused to a short E8 peptide generate E8^E2C proteins that are major repressors of viral expression and replication, which prevents runaway replication [44,45]. 

The E4 open reading frame is expressed as fusion proteins with a short peptide from the beginning of the E1 ORF to encode E1^E4 proteins that may facilitate virion egress [13,46,47]. E5 is a small membrane protein that regulates EGFR activity [11,12]. The L1 and L2 open reading frames encode the capsid proteins and are synthesized simultaneously with productive viral replication, leading to the encapsidation of newly replicated viral genomes into progeny virions [48,49]. 

## 3. Differentiation-Dependent Viral Life Cycle

HPV virions infect cells in the basal layer of stratified squamous epithelia that become exposed upon some form of wounding. Following entry, viral genomes migrate to the nucleus where they are established as low-copy episomes. Early transcripts encoding E6, E7, E1 and E2 are expressed resulting in the establishment of viral genomes at copy numbers of about 50 to 100 per cell [50]. These copy numbers are stably maintained in infected basal cells throughout the course of productive viral infection or until cleared by the immune response. In some cases, infections persist and progress to cancers. In cancers, HPV genomes are often found integrated in host chromosomes in a manner that retains expression of E6 and E7 but prevents that of E1 and E2 [51,52]. Replication of viral episomes in infected basal cells occurs in S-phase and is mediated by cellular polymerases and associated factors in coordination with chromosomal replication. After DNA replication is completed, viral genomes are distributed equally to each of the two daughter cells. One daughter cell remains in the basal layer and continues to actively divide while the other migrates toward the suprabasal layers and begins to differentiate [50,53,54]. HPV proteins only modestly alter cellular differentiation patterns and retain expression of markers such as keratin 10, filaggrin and loricrin in suprabasal layers [55]. It is not until lesions progress to cancer that the expression of these differentiation markers is lost. While normal cells exit the active cell cycle and enter G0 as they leave the basal layer, HPV-positive cells remain arrested in G1 until they reach the upper stratified layers. At this stage, HPV-positive cells re-enter S-phase, resulting in aberrant cellular replication, and transit into G2/M, where viral genome amplification and virion assembly occur [53]. Viral amplification in differentiated cells does not occur in S-phase but rather in G2/M because, as will be discussed below, it is dependent upon the activity of the homologous recombination repair arm of the DNA damage response that takes place in G2/M. Amplification occurs coordinately with expression of viral late transcripts that initiate from a promoter located in the E7 ORF leading to high-level expression of E2, E1, E1^E4 and E5 [50]. In addition, late transcripts encoding L1 and L2 are generated from the late promoter but require suppression of early polyadenylation signals to utilize termination elements at the end of L1 (Figure 1). This occurs through virally induced changes in the polyadenylation factor, CSTF-64 [56]. The synthesis of capsid proteins occurs coordinately with amplification resulting in the packaging of viral genomes into newly assembled virions for release from the exterior-most layers.

## 4. Activation of DNA Damage Repair Pathways in HPV-Positive Cells

HPV replication including differentiation-dependent amplification is mediated in large part by cellular enzymes whose activities have been hijacked by viral proteins. This includes polymerases and single-strand DNA binding proteins among others. Recent evidence indicates that HPV proteins activate members of the DNA damage repair pathways (DDR), and this is critical for viral replication [50]. All cells regularly acquire DNA breaks necessitating the low-level activation of DDR pathways; however, activation of these pathways is significantly enhanced in HPV-positive cells. Several DNA repair pathways with overlapping activities are responsible for the repair of both single-strand and double-strand breaks [57]. The non-homologous end joining pathway along with homologous recombination repair mediates most repairs. The primary factors regulating repair are members of the phosphatidylinositol 3-kinase-like protein kinase family (PIKK). This includes the ataxia-telangiectasia mutated (ATM), ataxia telangiectasia and RAD3-related (ATR) and the DNA-dependent protein kinase catalytic subunit (DNA-PKcs) enzymes [58]. ATM and DNA-PK are activated by double-strand breaks induced by endogenous or exogenous mechanisms such as ionizing radiation [59]. ATR is activated by single-strand DNAs that become exposed during double-strand break repair or from stalled replication forks [60,61]. Activation of both ATM and ATR is needed for HPV replication [62].

**Figure 2:**
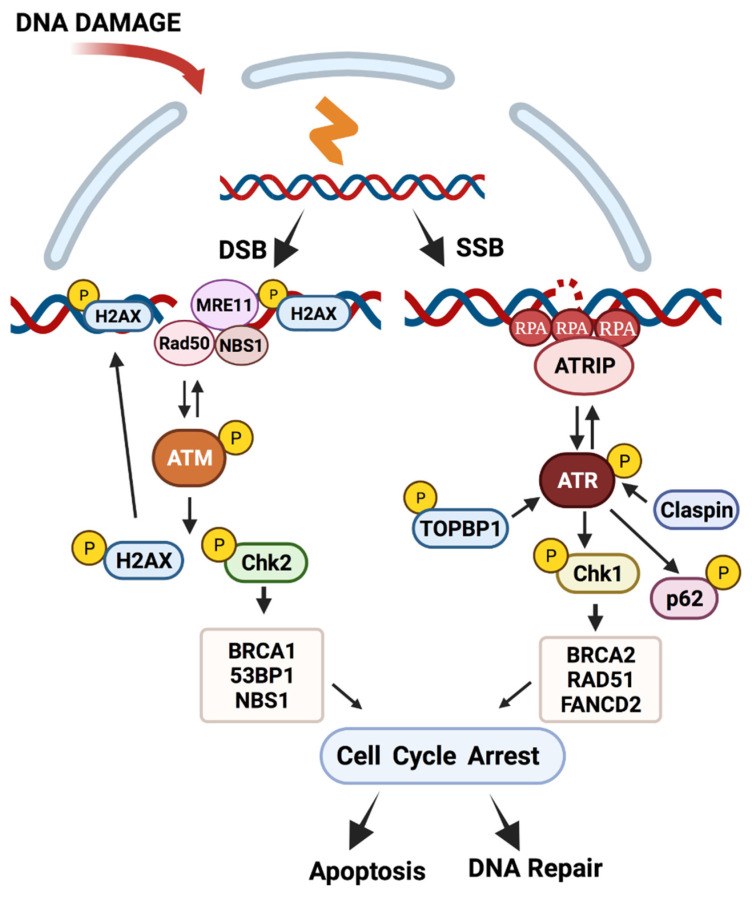
DNA damage repair pathways. The ATM and ATR DNA damage repair pathways are activated in response to dsDNA or ssDNA breaks/stalled replication forks, respectively. Activated ATM phosphorylates effectors such as CHK2, leading to the recruitment and phosphorylation of a series of DNA repair factors and cell cycle checkpoint arrest. Similarly, activated ATR phosphorylates effector protein CHK1, leading to activation of additional downstream factors. Both pathways mediate DNA repair through homologous recombination or otherwise eliminate the damaged cell by apoptosis or senescence. (Created with BioRender.com, 24 June 2021).

ATM proteins are activated through autophosphorylation following recruitment to sites of double-strand breaks by the MRN (Mre11–Rad50–NBS1) complex of proteins (Figure 2). Activation of ATM leads to the phosphorylation of a large number of downstream effectors and among these are the checkpoint kinase 2 (pCHK2) as well as the histone H2AX (γH2AX) [63]. Activated CHK2 can induce a checkpoint arrest in G2/M to allow for DNA repair or induce apoptosis. Major downstream targets of pCHK2 are 53BP1, NBS1 and BRCA1 [58]. Following activation by ATM, γH2AX binds to regions of up to several hundred kilobases that flank the DNA break and acts to mark these lesions. ATR is activated by complex formation with ATRIP, TOPBP1 and claspin, resulting in recruitment to sites of DNA break repair or long regions of single-stranded DNAs [60,61] (Figure 2). pATR induces phosphorylation of the checkpoint kinase 1 (pCHK1), which acts similarly to CHK2 in causing cell cycle arrest in G2/M or apoptosis [64]. Major targets of pCHK1 include BRCA2, RAD51 and FANCD2 from the Fanconi anemia pathway [65].

The homologous recombination arm of DNA repair takes place in G2/M as it requires the presence of a sister chromatid as a template [66]. Following recruitment of ATM to the sites of double-strand breaks by the MRN complex and activation, it phosphorylates CHK2 and the nuclease CtIP, which together with Mre11 induce end resection. This results in the generation of regions of single-stranded DNA that are quickly coated by RPA. RAD51 then replaces RPA leading to formation of filaments that mediate strand invasion of the sister chromatid and repair by DNA polymerases. 

Replication fork arrest can also induce DNA breaks, which can occur through the generation of bulky DNA lesions as well as collisions of RNA polymerases with DNA polymerases, resulting in formation of trimeric RNA:DNA structures called R-loops [67]. R-loops contain an exposed region of single-strand DNA that activates the ATR pathway. ATR then activates CHK1 to induce cell cycle arrest as well as phosphorylation of a number of chromatin binding factors that mediate a restart of the stalled forks. This process is also dependent upon members of the Fanconi anemia pathway particularly FANCM [65,68]. Members of the Fanconi anemia pathway are also responsible for the resolution of interstrand crosslinks of DNA that also result in breaks. FANCM activation leads to ubiquitination of FANCD2, leading to breaks that are the repaired by the homologous recombination repair pathways. Both FANCD2 and FANCM regulate R-loop formation, which contributes to stalled replication forks and ATR activation [65,69,70]. 

HPV-positive cells constitutively activate these pathways to levels significantly higher than normal cells [62,71]. This includes activation of ATM and ATR as well as their downstream effectors including pCHK1, pCHK2, γH2AX and pBRCA1 among others (Table 1). Activation of these DNA repair factors leads to their recruitment to distinct nuclear foci whose number and size are enhanced in HPV-positive cells. Furthermore, these foci also contain replicating HPV genomes [72]. While knockdown of ATM blocks amplification, it has minimal effects on the stable maintenance of episomes in undifferentiated cells [73]. In contrast, knockdown of ATR inhibits stable maintenance replication as well as amplification [74]. Activation of these two pathways in HPV-positive cells is not dependent on the presence of viral episomes but rather is mediated by the action of the E6 and E7 oncoproteins alone. Interestingly, overexpression of E1 from heterologous promoters also results in enhanced activation of DDR pathways, but whether this occurs when E1 is expressed at low levels from viral episomes is still unclear [54,71]. The E6 and E7 oncoproteins activate both ATM and ATR pathways (Table 1) by inducing very high levels of breaks in both cellular and viral sequences [75]. The breaks in viral episomes are rapidly repaired through the preferential recruitment of homologous recombination repair factors such as RAD51 and BRCA1 to viral genomes in differentiated cells, which leads to genome amplification [76].

The cohesin protein, SMC1, functions not only in facilitating pairing of sister chromatids in G2/M but is also critical in DNA repair, allowing for the recruitment of DDR factors. SMC1 also forms complexes with the insulator protein CTCF to regulate the formation of large DNA loops called topologically associating domains or TADs that regulate both transcription and replication [77].These domains are insulated from each other, allowing actively transcribed regions to be separated from silent regions. Replication or transcription in these TADs results in significant torsional stress that is relieved by another member of the SMC1/CTCF complex, topoisomerase 2β (TOP2β) [78]. TOP2β induces double-strand breaks, which allows for relieving torsional stresses by forming a covalent intermediate with the cleaved DNA while strand passage occurs. Failure to quickly resolve these protein:DNA intermediates results in DNA breaks that are repaired by DDR pathways. SMC1 is activated by HPV and in complex with CTCF is recruited to viral genomes. Depletion of SMC1 or CTCF leads to the inhibition of viral amplification [79]. In addition, the levels of TOP2β are significantly increased in HPV-positive cells and knockdown blocks HPV replication. TOP2β regulates HPV replication primarily through its role in DNA break formation as over half the DNA breaks in HPV-positive cells are due to TOP2β. Furthermore, TOP2β-induced DNA breaks are responsible for the activation of DDR pathways in HPV-positive cells [80]. 

DNA topoisomerase II beta-binding protein 1 (TopBP1) is another protein involved in regulating ATR activation and HPV replication. TopBP1 binds and helps to activate ATR in addition to being a target of ATR phosphorylation. Phosphorylation of TopBP1 by ATR activates its ability to act as a transcription factor that regulates the expression of several DNA damage repair factors along with E2F1 and p73. TopBP1 levels are increased in HPV-positive cells by the immune signaling protein STAT5 through the action of E7 [74]. In addition, E2 forms complexes with TopBP1, and this is important for the maintenance of viral episomes as well as for amplification [81]. Furthermore, ATR also phosphorylates p62, a critical cargo protein in autophagy, leading to suppression of GATA4 levels and downregulation of expression of a number of proinflammatory host genes [82] as well as the innate immune response. Importantly, knockdown of p62 or overexpression of GATA4 blocks the maintenance of viral episomes. These data suggest a linkage between the innate immune response and activation of DNA damage repair pathways in HPV-positive cells. 

## 5. HPVs Alter Innate Immune Pathways

To establish a persistent infection in keratinocytes, HPVs need to escape surveillance by the innate immune response, which is a critical host defense mechanism that has evolved as the first line of protection against pathogens. The innate immune response consists of a variety of sensors, referred to as pattern recognition receptors (PRRs), that detect the presence of foreign components such as bacterial lipopolysaccharides or viral nucleic acids such as cyclic nucleotides, ssRNA or dsRNA, cytosolic dsDNA and RNA-DNA hybrids [83]. Detection of these pathogen-associated molecular patterns (PAMPs) initiates a signaling cascade that results in the production of cytokines, such as interferons (IFNs), and the activation of hundreds of interferon-stimulated genes (ISGs), collectively creating a cellular “antiviral state”. The innate immune pathways can target viral infections at different stages ranging from entry to replication and release. Since HPVs can establish persistent infections that have the potential to develop into cancer, they have evolved distinct mechanisms for long-term suppression of immune recognition pathways. 

### 5.1. Sensors of Innate Immunity

HPVs interfere with several PRRs that are either membrane bound or cytosolic. Toll-like receptors (TLRs) are membrane-bound sensors that have been shown to be important in activating both innate and adaptive immunity [84]. HPV16 E6 and E7 have been identified as suppressors of TLR9 expression and function in keratinocytes through the recruitment of HDAC1 and JARID1B to the TLR9 promoter [85]. In a similar manner, the expression of TLR3 has been reported to be repressed in keratinocytes infected by hr-HPVs [86]. Interestingly, studies looking at biomarkers that can potentially predict HPV clearance have identified high levels of expression of TLRs: 3, 7, 8, 9 to be linked with viral clearance in patients [87,88]. Altogether, these studies suggest that TLRs have a significant role in regulating HPV infections. 

**Figure 3:**
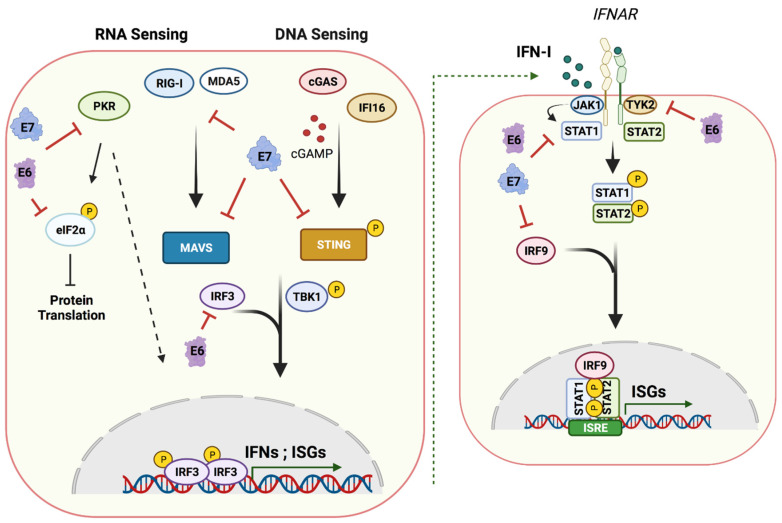
Host nucleic acid sensing pathways and targets of E6 and E7. A cartoon showing cytoplasmic nucleic acid sensors and their signaling cascades inducing type I IFN production and ISG expression that are targeted by E6 and E7 (left panel). A cartoon showing the effects of secreted IFNs on neighboring cells through activation of the JAK–STAT pathway leading to an antiviral state (right). HPV oncoproteins E6 and E7 manipulate these pathways at different stages, damping the innate immune response and establishing a persistent infection. (Created with BioRender.com, 24 June 2021).

HPVs have also been shown to target cytosolic DNA sensors of the innate immune pathways (Figure 3). When overexpressed, the interferon-gamma-inducible protein 16 (IFI16) DNA sensor can inhibit HPV18 replication and transcription [89]. Similarly, depletion of IFI16 in keratinocytes results in higher viral loads [90]. The absent in melanoma 2 or AIM2 is a PRR that is part of the inflammasome and is activated by HPV16 leading to enhanced production of IL-1β and IL-18 [91]. Interestingly, both IFI16 and AIM2 have been shown to be upregulated in HPV-positive cervical biopsies as well as head and neck carcinomas [91,92]. The recently identified sensor cyclic GMP-AMP synthetase (cGAS) [93,94,95] is another target of HPVs. HPV has been reported to silence cGAS signaling during viral entry through its effects on L2-dependent vesicular trafficking [96]. It remains controversial what effect persistent HPV infections have on the cGAS pathway as some reports suggest repression, while others increased expression. 

RNA sensors such as dsRNA protein kinase (PKR), retinoic-acid-inducible gene I (RIG-I) and MDA5 have also been identified as targets of HPVs. The levels of RIG-I and MDA5 transcripts in keratinocytes infected with hr-HPVs were found to be significantly reduced [86]. RIG-I signaling was also disrupted by E6 binding to TRIM25 and USP15, two upstream regulators of RIG-I [97]. In addition, E7 has been reported to induce epigenetic silencing of RIG-I and cGAS [98]. In a similar manner, cells containing either HPV16 or 31 genomes exhibited a reduction of PKR transcripts along with reduced levels of active, phosphorylated levels of PKR proteins that were mediated by E6 and E7 through post-transcriptional mechanisms [99]. PKR controls protein translation through eIF2α phosphorylation and is a critical regulator of antiviral immunity as well as maintenance of cell homeostasis. E6 binding to the GADD34/PP1 phosphatase complex directly inhibits phosphorylation of eIF2α [100], while HPV16 E6 relocates it to cytoplasmic clusters that co-localize with P-bodies [99].

### 5.2. Adaptors and Signaling Proteins

Adaptor proteins link the signaling from cytoplasmic sensors to the nucleus to initiate an innate immune response against pathogens (Figure 3). The stimulator of interferon genes (STING) serves as a signaling adaptor for IFI16 and cGAS. Upon binding of cGAS to dsDNA, cyclic GMP-AMP (cGAMP), a second messenger, gets synthesized [93,95,101,102]. cGAMP is recognized by and binds STING, leading to its activation [94,103]. STING is initially localized to the ER, and following activation by cGAMP, it migrates to the Golgi where it binds TBK1, leading to its autophosphorylation along with phosphorylation of the interferon regulatory factor IRF3. Activated IRF3 finally translocates to the nucleus to induce IFN and ISGs expression [104]. STING is also a downstream mediator of IFI16 signaling [89]. Given the importance of STING in signaling to the nucleus, it is targeted by many viruses including HPV. The E2 protein has been reported to suppress STING expression along with that of many other innate immune proteins [105]. The E7 protein also alters STING expression through epigenetic silencing [98]. Furthermore, HPV18 E7 has been reported to bind STING through its LXCXE motif, resulting in inhibition of its function [106]. Recent work in oropharyngeal carcinomas showed that depletion of E7 can restore cGAS–STING signaling [107]. Additionally, E6 of HPV16 binds directly to IRF3, hence possibly interfering with its transcription activation in the nucleus [108]. It appears that there are many ways HPVs interact with the cGAS–STING pathway, and the details of these mechanisms remain to be fully determined. 

### 5.3. IFN Signaling and ISGs

The endpoint of these innate signaling pathways is ISGs expression and IFN production. IFNs are classified into three types according to their sequence homology and to the receptors they signal through. IFN production and ISG expression provide two major functions: the first is to clear the viral infection and/or eliminate the infected cell; the second is to signal neighboring cells through secreted IFNs by initiating an antiviral program. Upon binding of IFNs to receptors on the surface of neighboring cells, Janus kinases (JAKs), which are bound to the cytoplasmic tails of receptors, become activated and phosphorylate a series of inactive transcriptional activators called STATs that are found in the cytoplasm. Phosphorylated STATs (predominantly STAT1 and STAT2) translocate to the nucleus to bind ISG promoter regions to activate transcription (Figure 3). Given the critical role of JAK–STAT signaling in controlling the interferon response to viral infections, it is targeted by HPVs in several ways (Figure 3). The transcription of STAT1 along with that of many other ISGs is silenced by both E6 and E7 oncoproteins [109,110]. If STAT1 levels are restored in HPV-positive cells using heterologous expression vectors, episomal templates are significantly reduced, and amplification is blocked [111]. Examination of STAT1 levels in cervical squamous carcinomas biopsies indicated a direct correlation between viral load and stage of disease [112]. Together, these studies confirm the overall importance of downmodulation of STAT1 by HPV proteins. 

In contrast to STAT1, HPVs appear to activate STAT5 and STAT3, which have been implicated in the development of a number of solid cancers (reviewed in detail in [113]). HPV E7 induces STAT5 phosphorylation, and this is important for high-level activation of ATM and ATR DNA repair pathways [114]. Transcription factor KLF13 was identified as responsible for increased STAT5 levels during HPV infection. Depletion of KLF13 correlated with decreased STAT5 expression along with impaired DDR activation and genome amplification [115]. Similarly, STAT3 phosphorylation is activated by HPV proteins. Activation of STAT3 is linked to enhanced cell proliferation along with inhibition of differentiation leading to immortalization [116]. Furthermore, cervical cancer tissues positive for HPV display high levels of activated STAT3 when compared to normal tissues [117]. In addition, E6 induces activation of the transcription factor NFκB, resulting in IL-6 production leading to STAT3 phosphorylation and nuclear localization [118,119]. Altogether, these studies emphasize the ability of HPVs to target different members of the STAT family leading to immune evasion and enhanced proliferation. 

HPVs suppress the expression of IFN-α and IFN-β through interference with STATs and binding kinase Tyk2 [120]. Keratinocytes express a specific type of IFN known as IFNκ, with κ standing for keratinocyte, that is constitutively expressed at low levels and is upregulated following viral infection [121]. HPV E6 and E7 suppress IFNκ expression through epigenetic silencing as well as through p62-mediated suppression of GATA4 expression. Restoring IFNκ expression in HPV-positive keratinocytes results in increased levels of IFN-regulatory factors and p53 [122]. Furthermore, E2 and E5 also act to suppress IFNκ and STAT1 levels [105,123,124]. Importantly, treatment of HPV-positive cell lines with IFNκ inhibited viral replication and cell growth, further demonstrating the importance of suppressing this interferon upon viral infection [125]. Hr-HPVs also target the interferon-related developmental regulator 1 by increasing its expression in an EGFR-dependent manner and preventing NFκB activation, leading to limited cytokine production [126]. The manipulation of IFN signaling by HPVs subsequently leads to the suppression of ISG expression. Analysis of patient biopsies and cell lines has identified Mx1, IFI56, OAS, ISG15, IFIT1, IFITM, RIG-I and MDA5 as prominent ISGs whose expression is suppressed by HPV proteins [82,86,109,125,127,128]. Most of the studies on IFNs and ISGs have been performed on cells with established HPV infections, and the events that occur during viral entry are not yet fully understood. 

## 6. DAMPs Connect DNA Damage and Innate Immunity during HPV Infection

The innate immune response is activated in response not only to foreign nucleic acids but also to “misplaced” self-DNAs that arise during periods of DNA damage. These damage-associated molecular patterns or DAMPs are markers of cellular malfunction and are detected by innate immune sensors that can lead to programed cell death. Genotoxic stress induces genomic instability that causes DNA breaks. These broken DNA fragments can leak into the cytosol, where they are recognized by the PPRs. Sensors such as cGAS, IFI16 and RIG-I but also STING have been linked to genomic instability [129,130,131,132,133]. This crosstalk between genomic instability, DNA repair and innate immunity is critical in maintaining cell homeostasis, and currently there is an increased interest in the use of immune nucleic acid sensing pathways in targeting cancer [134]. 

As described in detail previously, HPV oncoproteins induce DNA breaks in infected cells to activate DDR mechanisms, which are important for viral amplification. A linkage between the DNA damage response and innate immunity in HPV-positive cells is suggested by studies linking ATR activation with phosphorylation of the autophagy protein p62, which in turn acts to suppress the transcription factor GATA4. This results in the suppression of cytokines such as IL-5 and CXCL2 and IFNκ expression [82]. Ongoing studies indicate cGAS may act to inhibit DNA repair in HPV-positive cells in a STING-independent manner. In response to DNA damage, cGAS translocates to the nucleus, where it associates with chromatin to downregulate homologous recombination repair [135]. Additional reports suggest cGAS localizes to micronuclei upon DNA damage [136]. The genomic instability caused by HPV could be a successful target for efficient therapeutics through innate immune activation, hence preventing malignant progression. For instance, ATR inhibitors have been used to treat HPV-positive oropharyngeal cancers, but the morbidity of these treatments remains high. Inclusion of activators of the innate immune response may make treatments more effective and reduce the side effects. In conclusion, crosstalk between DNA damage repair pathways and innate immune signaling may affect critical cellular processes such as autophagy, senescence and apoptosis during HPV infections. 

## 7. Conclusions 

In this review article, we have described the current knowledge of two major pathways used by hr-HPVs to establish long-term persistent infections. HPVs activate host DNA damage repair pathways to allow for genome amplification upon differentiation as well as to manipulate the innate immune responses to avoid clearance. Furthermore, it appears that these two pathways are interlinked, and HPVs need to target both for a successful infection. These studies describe biomarkers that may facilitate prognosis as well as novel ways to efficiently target and treat HPV-positive anogenital and oropharyngeal cancers. 

## Figures and Tables

**Table 1:** DNA damage repair factors altered by HPV oncoproteins ATM and ATR DNA damage repair factors that are activated or increased in levels through the action of E6 and E7 oncoproteins.

ATM Targets	ATR Targets
ATM, MRE11, RAD50, NBS1, Chk2, BRCA1, RAD51, 53BP1, H2AX, RNF168, Tip60, SMC1	ATR, ATRIP, Chk1, TopBP1, FANCD2, FANCM, BRCA1, BRCA2, RAD51, p62, H2AX

## Data Availability

Not Applicable.
